# Early extubation is not associated with severe intraventricular hemorrhage in preterm infants born before 29 weeks of gestation. Results of an EPIPAGE-2 cohort study

**DOI:** 10.1371/journal.pone.0214232

**Published:** 2019-04-04

**Authors:** Marie Chevallier, Pierre-Yves Ancel, Héloïse Torchin, Laetitia Marchand-Martin, Elsa Lorthe, Patrick Truffert, Pierre Henri Jarreau, Jean Christophe Roze, Véronique Pierrat, Stéphane Marret, Olivier Baud, Valérie Benhammou, Anne Ego, Thierry Debillon

**Affiliations:** 1 UMR 5525 ThEMAS, CNRS, TIMC-IMAG, Grenoble Alpes University, Grenoble, France; 2 Neonatal Intensive Care Unit, Grenoble Alpes University Hospital, Grenoble, France; 3 Obstetrical, Perinatal, and Pediatric Epidemiology ResearchTeam (Epopé), INSERM UMR 1153, Center for Epidemiology and Biostatistics Sorbonne Paris Cité; Paris Descartes University, Paris, France; 4 Risk in Pregnancy DHU, Cochin Hotel-Dieu Hospital, APHP, Paris, France; 5 EPIUnit–Institute of Public Health, University of Porto, Porto, Portugal; 6 Neonatal Intensive Care Unit, Jeanne de Flandre Hospital, CHRU Lille, Lille, France; 7 Neonatal Intensive Care Unit Port-Royal, Hôpital Cochin, APHP Paris, France; 8 Department of Neonatal Medicine, CHU Nantes, Nantes, France; 9 Department of Neonatal Medicine and Intensive care, CHU Rouen, Rouen, France; 10 Division of Neonatology and Pediatric Intensive Care, CHU Geneva, Geneva, Switzerland; Johns Hopkins School of Public Health, UNITED STATES

## Abstract

**Objective:**

To determine whether there is an association between severe intraventricular hemorrhage and early extubation in preterm infants born before 29 weeks of gestational age and intubated at birth.

**Methods:**

This study included 1587 preterm infants from a nationwide French population cohort (EPIPAGE-2). Secondary data on intubated preterm infants were analyzed. After gestational age and propensity score matching (1:1) we built two comparable groups: an early extubation group and a delayed extubation group. Each neonate in one group was paired with a neonate in the other group having the same propensity score and gestational age. Early extubation was defined as extubation within 48 hours of life. Severe intraventricular hemorrhages were defined as grade III or IV hemorrhages according to the Papile classification.

**Results:**

After matching, there were 398 neonates in each group. Using a generalized estimating equation model, we found that intraventricular hemorrhage was not associated with early extubation (adjusted OR 0.9, 95%CI 0.6–1.4). This result was supported by sensitivity analyses.

**Conclusion:**

The practice of early extubation was not associated with an increased proportion of intraventricular hemorrhages. To complete these results, the long-term neurologic outcomes of these infants need to be assessed.

## Introduction

In very preterm infants (<32 weeks gestation), short and long-term pulmonary and neurological outcomes are major concerns. Severe bronchopulmonary dysplasia (BPD) is diagnosed in 13–32% of these infants.[1,2] However, mechanical ventilation (MV) presents major risks as it can lead to chronic inflammation.[3–5] Consequently, offering adapted gentle ventilation in order to preserve immature lungs is a daily challenge for neonatologists.[6] In 2016, the European guidelines strongly recommended minimizing the duration of MV in preterm infants. Additionally, the current trend in neonatal intensive care units (NICU) is to extubate these infants using several different strategies. For example, the InSurE procedure (Intubation-Surfactant-Extubation)[7] generally comprises extubation within 1 hour after intubating the neonate in order to administer surfactant. However, the effects of rapid extubation on brain injury have been little studied.[8] In some cases, physicians wait several hours before extubating the neonate, because extubation during the so-called transitional period (before 48–72 hours of life) [9,10] exposes very preterm infants to higher risks of respiratory and haemodynamic instability, which could be prejudicial for immature brains.

We asked whether extubating these neonates during this period could have neurological consequences, especially in the event of severe intraventricular hemorrhage (IVH) (grade III or IV).[11] Most IVHs occur in the first week of life[12] and in the literature are associated with low superior vena cava flow[13], symptomatic low blood pressure[14], abnormal regional cerebral oxygenation during apnea [15,16], low pH and hypocapnia.[17] Early extubation (EE) could be associated with some of the risk factors for severe IVH, especially apnea, bradycardia or hypercarbia. For this reason, we considered it important to explore whether EE within 48h of birth is associated with an increased risk of IVH. Severe IVH occurs in 5–12% of very preterm infants [1] and about one-third of these will subsequently develop cerebral palsy.[18–20] Due to the widespread implementation of reduced duration MV, despite the lack of studies on its effect on IVH, the assumption that early extubation (EE) within 48h of birth is not associated with increased risk of IVH needs to be explored. The timing of extubation is dependent on multiple factors making it difficult to select a homogeneous population for a randomized control trial (RCT). Thus, large observational studies, complementary to RCTs, are needed to evaluate the impact of EE on IVH.

Based on EPIPAGE-2, a large prospective French population based cohort that included very and extremely preterm infants born in France in 2011, our objective was to evaluate the association between IVH and EE in neonates born before 29 weeks gestational age (GA) who were intubated at birth.

## Patients and methods

### Study population

The prospective population-based cohort EPIPAGE-2 included all births occurring between 22 and 31^6^ weeks’ gestation in 25 French regions, in 2011. Inclusion and data collection occurred only after families had received information and orally agreed to participate in the study. The EPIPAGE-2 study was approved by the National Data Protection Authority and ethics committees (Comité consultatif sur le traitement de l'information en matière de recherche dans le domaine de la santé, Comité de protection des personnes Ile-de-France); details about the design and methods are described elsewhere [21]. The EPIPAGE-2 cohort study collected detailed maternal and neonatal data including cardiovascular, neurological, respiratory, and nutritional parameters, and follow-up of survivors. For the present database study, we analyzed secondarily respiratory and neurological data of neonates born alive between 24 and 28^6^ weeks’ gestation, who were intubated in the delivery room and then admitted to the NICU. Newborns with severe congenital malformations (n = 29) were not included. Death within 48 hours of birth (n = 36) or nitric oxide (NO) administration within 72 hours of birth (n = 88) were strong markers of a severe respiratory status precluding EE; therefore, these neonates were not included. In addition, neonates with missing data on cranial ultrasound (cUS) (n = 2) or extubation timing (n = 17) were not retained. Data concerning perinatal care were collected prospectively during the NICU stay via detailed standardized questionnaires. All data were fully anonymized before the analysis.

### Exposure of interest: Extubation within 48 hours of life

Information on initial respiratory management included intubation in the delivery room, date of first and definitive extubation, age at first, second and third doses of surfactant and duration of MV. Neonates were classed into two groups, according to whether or not they had EE. Those extubated at ≤48 hours of life were classed in the “Early Extubation” (EE) group; and neonates extubated at >48h were classed in the “Delayed Extubation” (DE) group. All extubation attempts were considered whether or not infants were re-intubated.

### Outcome: Intraventricular hemorrhage

To identify cerebral lesions, the standard practice in France is to perform 1 or 2 cUS examinations during the first week after birth (generally in the first 48h) and then weekly for the following 2 weeks. Data on abnormalities detected by cUS were collected using detailed standardized questionnaires. The Papile classification was used to rank IVH, and the Levene index was used to diagnose ventricular dilatation. [22,23] Before analysis, a panel of neonatologists (M.C., V.P., S.M., and T.D.) reviewed all diagnosed grade III and IV IVH cases, along with other available data, and confirmed the consistency of the cUS data for each.

### Other data analyzed

Gestational age was the best estimate based on a combination of last menstrual period and ultrasound assessment. We used customized growth reference curves to identify severely small-for-gestational-age (SGA) newborns (birthweight <3^rd^ percentile).[24] Other data collected included: mode of delivery (vaginal or C-section), context of birth (preeclampsia, preterm labor, preterm premature rupture of membranes, abruptio placenta, etc.), antenatal steroid and magnesium sulfate administration, level of neonatal care available in the hospital[25], sex, Apgar score, NO administration, chest compression in delivery room, patent ductus arteriosus (PDA) treated by ibuprofen during the first 48 hours of life, and vasoactive agents within the first 72 hours of life.

### Statistical analysis

For analyses of the study cohort, we used weighted percentages and adjustment for GA to take into account the differences in the recruitment times for infants born at 24 to 26^6^ weeks and those born at 27 to 28^6^ weeks ‘gestation. Medians were compared using the Wilcoxon test and means compared using a Student’s t-test.

### Propensity score

As the circumstances of birth and the severity of the infant’s condition could influence the possibility of EE and neurological outcomes, we used a propensity score method to account for observed confounding factors. The propensity score was defined as the infants’ probability of being extubated within the first 48 hours of life, based on individual covariates. Variables used in a propensity score should be more closely related to the outcome (IVH) than to the exposure (EE) [26]. The variables used in our study, were chosen because according to the literature they are associated with IVH, clinically relevant and/or associated with the outcome in the univariate analysis. The propensity score was estimated using a logistic regression model with EE as the dependent variable using the following baseline characteristics: GA, sex, antenatal magnesium sulfate and corticosteroid treatment, mode of delivery, birth in a context of abruptio placenta, maternity unit level of care, Apgar score <7 at 5 min of life, chest compression in delivery room, and number of surfactant doses. The proportion of neonates with missing data ranged from 0 to 10%. Missing data were treated as a separate category in the propensity score when these reached 2% of infants in the studied group. We used a 1:1 matching algorithm (without replacement) to match exposed (EE) and unexposed (DE) newborns on propensity score and GA with a caliper of width measuring the standard deviation of the score’s logit (0.2).[26] The balance of the match was analyzed using standardized differences. Groups were considered balanced if the standardized difference for each variable was less than 10% between the two groups.[26] After matching, a logistic regression model (Model 0) was applied using a generalized estimating equation (GEE) to account for paired data (matched cohort) and the average treatment effect assessed. ORs were calculated with a 95% confidence interval (95%CI).

Two sensitivity analyses were performed. The first (Model 1) was based on the propensity score-matched cohort using GEE logistic regression models. Model 1 variables, “need for vasoactive treatment within 72 hours” and “ibuprofen treatment for PDA during first 48 hours of life”, were considered as additional markers of severity and were added to the propensity score. The second sensitivity analysis (Model 2) was conducted on the study cohort, with a GEE logistic regression model to consider the hierarchical structure of infants clustered in neonatal care units because extubation practices could differ between centers. This model was adjusted on the same factors as those included in the propensity score for the main analysis. STATA SE 13 was used to perform all analyses.[27]

## Results

**Fig 1:**
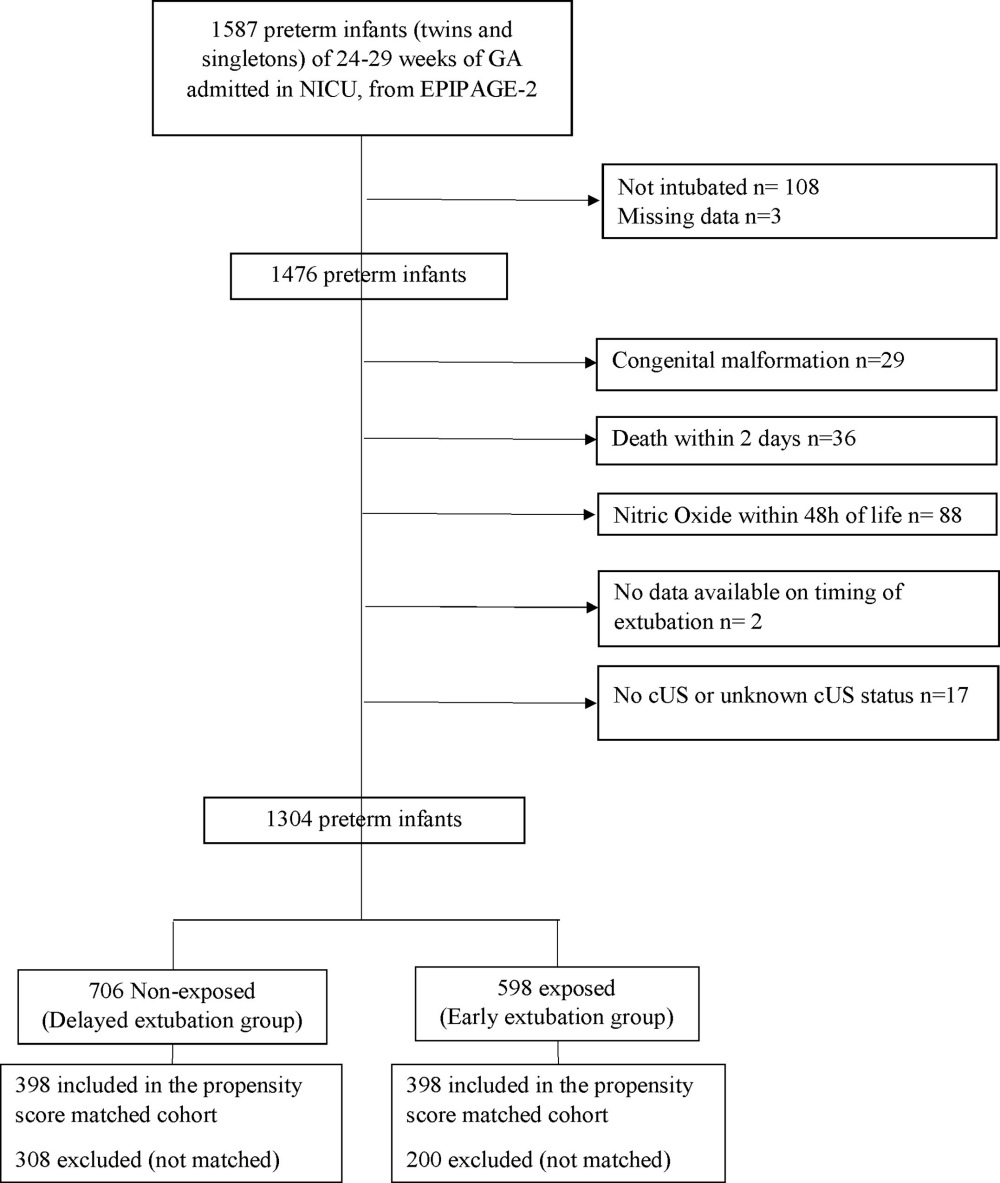
Study flow chart.

During the study period, from March 28 to December 31, of the 1587 preterm infants (twins and singletons) born in France between 24–28^+6^ weeks GA and included in the EPIPAGE-2 cohort, 1476 were intubated and eligible for the present study (Fig 1). The intubation rate varied between 86% at 28 weeks GA and 98% at 24 and 25 weeks GA (S1 Table). Among neonates born under 29 weeks GA intubated at birth, 97% received surfactant including 2/3 in the delivery room (S1 Table).

After applying the exclusion criteria, the final study cohort was 1304 preterm infants, 706 in the DE group and 598 in the EE group. In the DE group, 16.4% of neonates had severe IVH vs 7.3% in the EE group. In the study cohort, the median [interquartile range] time to a first extubation attempt was 2 days [1–6]. We compared our population sample (n = 1304) to neonates excluded due to death within 48 hours or NO administration, or with missing data (n = 143) (S2 Table). Excluded preterm infants were younger, had a lower 5 min Apgar score and were extubated later.

### Characteristics of the study population

Tables 1 and 2 show the baseline characteristics of the study- and propensity score—matched cohorts according to EE or DE.

**Table 1:** Baseline variables according DE and EE within study cohort/ Propensity score-matched cohort.

	STUDY COHORT						PROPENSITY SCORE—MATCHED COHORT					
	Delayed extubation n = 706		Early extubation n = 598				Delayed extubation n = 398		Early Extubation n = 398			
	n	%*	n	%	p-value	SD^**^ *(%)*	n	%	n	%	p-value	SD^**^ *(%)*
**Gestational age**												
*24*	76	9.5	10	1.4	<0.05	*38*.*3*	10	2.5	10	2.5	1	*0*
*25*	156	19.5	70	9.7	0.1	*28*.*0*	70	17.6	70	17.6	1	*0*
*26*	186	23.2	144	19.9	0.6	*5*.*2*	113	28.4	113	28.4	1	*0*
*27*	145	24.1	170	31.4	0.4	*18*.*4*	99	24.9	99	24.9	1	*0*
*28*	143	23.8	204	37.6	0.1	*31*.*5*	106	26.6	106	26.6	1	*0*
**Sex**					0.9	*1*.*6*					0.2	*8*.*5*
*Male*	372	52.8	320	53.2			211	53.0	194	48.7		
*Female*	334	47.2	278	46.8			187	47.0	204	51.3		
**Mode of delivery**					0.5	*7*.*3*					0.1	*10*.*0*
*Vaginal*	287	39.8	253	40.9			165	41.5	133	34.2		
*C- section*	401	59.1	343	58.8			231	58.0	261	65.3		
*Missing data*	8	1.1	2	0.3			2	0.5	2	0.5		
**Antenatal steroids administration**					0.1	*3*.*5*					0.3	*2*.*2*
*Yes*	390	55.6	376	62.7			246	61.8	224	56.3		
*No*	286	40.1	205	34.5			140	35.2	158	39.7		
*Missing data*	30	4.2	17	2.8			12	3.0	16	4.0		
**Magnesium sulfate**					<0.05	*6*.*3*					0.5	*0*.*3*
*Yes*	46	6.6	61	10.3			29	7.3	21	5.3		
*No*	648	91.9	524	87.7			361	90.7	370	93.0		
*Missing data*	12	1.6	13	2.0			8	2.0	7	1.8		
**Category of birth unit**					0.2	*13*.*0*					0.8	*0*.*2*
*Level I*	28	4.2	21	3.5			18	4.5	17	4.3		
*Level II*	87	12.2	43	7.1			35	8.8	41	10.3		
*Level III*	591	83.6	534	89.4			345	86.7	340	85.4		
**Abruptio placenta**					0.7	*4*.*2*					0.9	*1*.*7*
*Yes*	39	5.8	34	5.8			21	5.5	22	5.3		
*No*	655	92.6	557	93.0			370	93.0	370	93.0		
*Missing data*	12	1.6	7	1.2			5	1.5	7	1.7		
**Chest compression**					0.1	*14*.*9*					1.0	*1*.*1*
*Yes*	64	9.0	30	4.7			27	6.8	28	7.0		
*No*	606	85.9	552	92.7			354	88.9	354	89.0		
*Missing data*	36	5.1	16	2.6			17	4.3	16	4.0		
**Apgar at 5min <7**					0.1	*9*.*9*					0.7	*1*.*0*
*Yes*	195	27.4	122	20.2			93	23.4	103	25.9		
*No*	443	63.2	430	72.4			270	67.8	260	65.3		
*Missing data*	68	9.4	46	7.4			35	8.8	35	8.8		

Percentages concerning study cohort are weighted according to gestational age

**Standardized difference

**Table 2:** Baseline variables according DE and EE within study cohort/ Propensity score-matched cohort.

		STUDY COHORT										PROPENSITY SCORE—MATCHED COHORT											
		Delayed extubation n = 706				Early extubation n = 598						Delayed extubation n = 398				Early Extubation n = 398							
		n		%*		n		%		p-value	SD^**^ (%)	n		%		n		%		p-value		SD^**^ *(%)*	
**Number of surfactant doses**										<0.05	*50*.*8*									0.1		*1*.*6*	
*0*		13		2.0		26		4.6				12		3.0		10		2.5					
*1*		366		51.9		487		81.5				300		75.4		303		76.1					
*2*		248		35.2		65		10.7				65		16.3		65		16.3					
*≥3*		72		10.0		17		2.8				17		4.3		17		4.3					
*Missing data*		7		0.9		3		0.4				4		1		3		0.8					
**Other variables not included in the propensity score**																							
**Birth weight z-score**										0.4	*4*.*8*									0.7		*2*.*7*	
*< 3*^*rd*^ *percentile n*, *%*		120		18.0		91		16.1				68		17.1		64		16.1					
*>3*^*rd*^ *percentile n*, *%*		586		82.0		507		83.9				330		82.9		334		83.9					
**PDA treated by ibuprofen within 48H**										0.1	*16*.*2*									0.13		*10*.*3*	
*Yes*, *n*, *%*		110		15.7		54		9.0				52		12.8		36		8.8					
*No*, *n*, *%*		581		82.3		539		90.3				343		86.2		361		90.7					
*Missing data*, *n*, *%*		15		2.0		5		0.7				4		1.0		2		0.5					
**Vasoactive agents within 72h**										<0.05	*16*.*6*									<0.05		*7*.*5*	
Yes, n, %		126		17.6		40		6.7				58		14.6		33		8.3					
No, n, %		570		81.0		553		92.4				337		84.7		362		90.9					
Missing data, n, %		10		1.4		5		0.8				3		0.7		3		0.8					

Percentages concerning study cohort are weighted according to gestational age

^*^ Standardized difference

### Study cohort

There was a significantly higher proportion of preterm infants born at 24 and 25 weeks GA in the DE group (9% and 19%, respectively) than in the EE group (1% and 10%, respectively). The frequency of complete antenatal steroid administration, infant’s sex, mode of delivery, and abruptio placenta were not significantly different between the two groups. The number of outborn infants and those who needed chest compression at birth was higher in the DE group (90% and 5%) vs (84 and 3%) in the EE group. A higher proportion of neonates received two doses of surfactant in the DE group vs the EE group (35% vs 11%, standardized difference = 50%). After univariate analysis, EE was found to be associated with a lower risk of IVH (OR 0.4, 95%CI 0.3–0.6) (Table 3).

**Table 3:** Severe IVH and early extubation.

	No of IVH/ No of patients		
Analysis method	DE n/N (%^a^)	EE n/N (%^a^)	OR [95% CI]
**Univariate analysis (study cohort)**	126/706 (16.4)	48/598 (7.4)	**0.4 [0.3–0.6]**
**Propensity score and GA matched cohort**			
Model 0	44/398 (11.0)	40/398 (10.0)	**0.9 [0.6–1.4]**
Model 1^b^	45/392 (11.5)	38/392 (9.7)	**0.8 [0.5–1.2]**
**Study cohort**			
Model 2^c^	**-**	**-**	**0.7 [0.5–1.1]**

^a^ Weighted percentage, concerning study cohort

^b^ data about ibuprofen administration for patent ductus arteriosus during 48H of life and vasoactive treatment were added to the propensity score

^c^ Logistic regression adjusted on the propensity score, fitted using generalized estimating equations to take account of non-independence of variables in the same hospital center.

### Matched cohort

After 1:1 matching on the propensity score and GA, we obtained two groups of 398 infants. The propensity score ranged from 0.02 to 0.83. The characteristics of both groups were well balanced in the matched cohort (standardized differences ≤10% between DE and EE groups), except for the mode of delivery for which the standardized difference was 10%, and could be considered as acceptable. The proportion of C-sections was higher in the DE group. Variables not included in the propensity score were PDA treatment within 48 hours and vasoactive agents within 72 hours; the standardized differences were ≤10%.

### Early Extubation and risk of Intraventricular Hemorrhage

In the main analysis with a propensity score-matched cohort, using a GEE model after adjustment for GA (model 0), EE was not associated with an increase in the rate of IVH (ORa 0.9, 95%CI 0.65–1.4). The proportion of neonates with severe IVH was the same in both groups (10% and 11%). The results were concordant in all sensitivity analyses (Table 3).

## Discussion

In our study cohort, newborns with DE were frailer than EE newborns. In univariate analysis EE was associated with a decreased risk of IVH. By rendering the two groups comparable through the use of a propensity score and GA matching, EE was found not to be associated with an increase in severe IVH in neonates born before 29 weeks GA. These results were consistent with the other models used in our study.

EPIPAGE-2 is a large prospective cohort, with an inclusion rate of almost 93% of preterm infants born before 29 weeks GA in France. To our knowledge, the present study is the first large prospective cohort study focusing on early extubation with IVH as primary outcome instead of severe bronchopulmonary dysplasia.[28,29] Various authors have shown that data from EPIPAGE-2 reflected French neonatal practices in 2011. The EPIPAGE-2 cohort has enabled studies on questions that had been debated in the literature and for which RCTs are difficult to design and conduct. [14] In this situation, it is difficult to suggest recommendations as to neonatal characteristics and the timing of extubation, particularly in view of the rare incidence of IVH. Our challenge was to build two groups (DE and EE), equivalent to the randomized groups of a RCT, with relatively large sample sizes.

To avoid potential selection bias where the frailest neonates were those most likely to be extubated late or to suffer IVH, a propensity score method was used. Although the propensity score method tries to reproduce RCT conditions, it does not take into account hidden or unknown factors [26]. In our primary analysis, we might have induced a selection bias by retaining only newborns who survived and did not have NO treatment in their first 72 hours of life, excluding those at higher risk of IVH because they were frailer. We performed another analysis (data not shown) that demonstrated that when newborns who had died or required NO <48h were included, the results did not change. While extubation practices could differ between centers, Model 2 took this into account, and the results were unchanged.

In the literature there is no consensus as to the definition of EE of preterm infants, and we acknowledge that our use of 48 hours of life as a cutoff is debatable. In a recent international survey of extubation practices, the first attempt to extubate was performed before 3 days of life in 76% of centers. [30] Berger et al. found that 38.5% of their cohort of neonates born <28 weeks GA had a first extubation at 1–3 days. [29] A strong reason for choosing the cutoff of 48 hours of life was that during the first two days the neonate is considered to be in a neurological transitional period with a high risk of cerebral blood flow changes. The ductus arteriosus is often not completely closed, and high pulmonary resistance can persist, weakening the newborn.[9] Finally, a 24 h delay in extubating was not studied with a sensitivity analysis because the percentage of neonates was too small (20%).

The proportion of neonates with severe IVH in the propensity score—matched cohort (approximately 10%) was similar to that described in the literature.[1] All cases of severe IVH were reviewed by an expert panel but we lacked information on the exact timing of the IVH, so we were unable to quantify the number of IVH cases diagnosed before early extubation. Severe IVH generally appears in the first 96 h of life. [31] There could be a problem concerning neonates with severe IVH whose neonatal physician prolonged intubation in the context of life-sustaining treatment, consequently slightly overestimating the number of IVH in the DE group.

In our study, the intubation rate was >90% in preterm infants born at 24−28^6^ weeks GA; this was higher than in previous studies of surfactant prophylaxis: 83% in the SUPPORT study [32] (24−27^6^ weeks GA) and 46% in the COIN study [33] (25−28^6^ weeks GA) in the “no prophylactic surfactant groups”. In our study, 60% of newborns received surfactants in the delivery room. These differences might reflect a higher rate of surfactant prophylaxis in neonates ≤28 weeks GA, which could be the result of a period in France when updated European guidelines (2010) were followed that did not recommend surfactant prophylaxis in neonates born at less than 27 weeks GA.[34] We might also ask if the management of respiratory distress has remained the same between 2011 and 2019. We suspect that early extubation is probably more frequent in 2019 than in 2011. In addition, we found a low rate of complete antenatal corticosteroid administration (59%), whereas currently, we know that this rate can be higher.[1] All these points could be limitations to generalizing our results to current practices.

In the EE group, the success rate of first extubation (defined by absence of reintubation until discharge) was almost 50% in our study. In the literature, this proportion varies between 50 and 70% depending on the GA and time defining extubation failure.[35,36] It is also questionable whether waiting to extubate is as safe as trying to extubate, especially in very immature preterms who are at a high risk for reintubation. In a retrospective study, Chawla et al. showed a higher proportion of severe IVH in infants with failed extubation.[35] In our study, the rate of severe IVH was not statistically significant (p = 0.3) between infants in the EE group who were reintubated (15.4%) and infants in the DE group who were reintubated (12.2%). Our results should be interpreted with caution as firstly we looked at extubation and IVH (following the first extubation attempt). We did not study the risk factors for IVH but focused on determining if EE was safe at 48 hours regardless of the success or failure of extubation. Secondly, the exact timing of reintubation, or the diagnosis of IVH and the cause of reintubation were not available in the EPIPAGE 2 database. This restricted the analysis because we could not know precisely which occurred first (extubation failure or IVH).

To date, no clear data regarding IVH and duration of MV have been published; the literature consists of retrospective studies and univariate data on this topic. One retrospective single center study that included 95 newborns showed a decreased rate of IVH in extremely low birthweight infants extubated within 48 hours of life. [37] Another study found an unadjusted risk of IVH in neonates with late or delayed extubation (at >3 days of life), but the authors were unable to determine whether the difference was a cause or a consequence [22]. Studies about InSurE or minimally invasive surfactant therapy (which could be considered as a very early extubation without MV) did not demonstrate a higher risk of IVH even after multiple InSurE attempts [28,29].

Finally, except for the pulmonary and cerebral benefits, extubating infants as soon as possible could be considered a part of developmental care and have a positive impact by reducing their length of stay in the NICU.

## Conclusion

In an era when gentle ventilation techniques are recommended, early extubation might be considered as aggressive and the practice questionable in very preterm infants, particularly during the neurological “transitional period”. Through the EPIPAGE-2 cohort, this study showed that in infants born between 24–29 weeks of GA, extubation within 48 hours of life is not associated with a higher frequency of IVH. This result encourages the practice of early extubation. Further prospective studies are needed to focus on the long-term neurological follow up of infants who were extubated early.

## Supporting information

S1 Table Percentage of infants born under 29 weeks of gestational age in the EPIPAGE 2 cohort who were intubated and who received surfactant. (TIF)

S2 Table Comparison of study cohort and excluded infants (NO, death, missing data). (TIF)

## Abbreviations

CI: confidence interval
cUS: cranial ultrasound
EE: early extubation
GA: gestational age
GEE: generalized estimating equation
InSurE: intubation surfactant extubation
IVH: intraventricular hemorrhage
DE: delayed extubation
MV: mechanical ventilation
NICU: neonatal intensive care unit
NO: nitric oxide
OR: odds ratio
PDA: patent ductus arteriosus
RCT: randomized controlled trial
SD: standardized difference
